# Crystal structure of (1*E*,1′*E*)-*N*,*N*′-(ethane-1,2-di­yl)bis­[(pyridin-2-yl)methanimine]

**DOI:** 10.1107/S2056989015010087

**Published:** 2015-05-30

**Authors:** Muneer Abdoh, Ismail Warad, S. Naveen, N. K. Lokanath, Rachid Salghi

**Affiliations:** a Department of Physics, Science College, An-Najah National University, PO Box 7, Nablus, Palestinian Territories; b Department of Chemistry, Science College, An-Najah National University, PO Box 7, Nablus, Palestinian Territories; c Institution of Excellence, University of Mysore, Manasagangotri, Mysore 570 006, India; d Department of Studies in Physics, University of Mysore, Manasagangotri, Mysore 570 006, India; e Laboratory of Environmental Engineering and Biotechnology, Science College, An-Najah National University, ENSA, Universite Ibn Zohr, PO Box 1136, 80000 Agadir, Morocco

**Keywords:** crystal structure, pyridine­carbaldehydes, 1,2-di­amino­pyridine, Schiff base, chelating ligands

## Abstract

The whole mol­ecule of the title compound, C_14_H_14_N_4_, is generated by twofold rotation symmetry. The twofold axis bis­ects the central –CH_2_-CH_2_– bond and the planes of the pyridine rings are inclined to one another by 65.60 (7)°. In the crystal, there are no significant inter­molecular inter­actions present.

## Related literature   

For the use of Schiff bases, derived from pyridine­carbaldehydes, in synthetic chemistry, see: Marjani *et al.* (2009 ▸). For 1,2-di­amino­pyridine-derived Schiff bases as bidentate or polydentate chelating ligands and their possible medical applications, see: Warad *et al.* (2014 ▸).
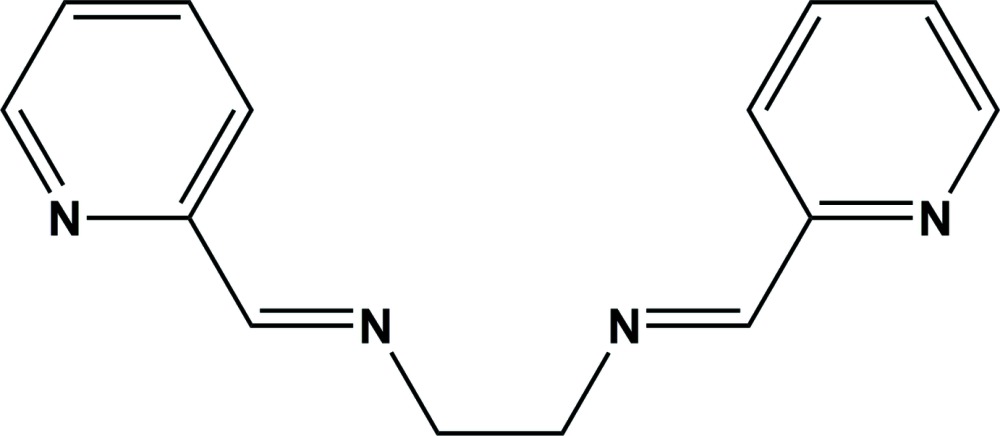


## Experimental   

### Crystal data  

C_14_H_14_N_4_
*M* *_r_* = 238.29
Monoclinic, 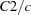

*a* = 19.347 (5) Å
*b* = 5.9339 (12) Å
*c* = 13.165 (2) Å
β = 122.266 (8)°
*V* = 1278.0 (5) Å^3^
*Z* = 4
Cu *K*α radiation
μ = 0.61 mm^−1^
*T* = 296 K
0.30 × 0.27 × 0.25 mm

### Data collection  

Bruker X8 Proteum diffractometer
Absorption correction: multi-scan (*SADABS*; Bruker, 2013 ▸) *T*_min_ = 0.837, *T*_max_ = 0.862
1539 measured reflections
933 independent reflections
881 reflections with *I* > 2σ(*I*)
*R*_int_ = 0.015

### Refinement  

*R*[*F* ^2^ > 2σ(*F* ^2^)] = 0.043
*wR*(*F* ^2^) = 0.120
*S* = 1.05
933 reflections
82 parameters
H-atom parameters constrained
Δρ_max_ = 0.10 e Å^−3^
Δρ_min_ = −0.10 e Å^−3^

### 

Data collection: *APEX2* (Bruker, 2013 ▸); cell refinement: *SAINT* (Bruker, 2013 ▸); data reduction: *SAINT*; program(s) used to solve structure: *SHELXS97* (Sheldrick, 2008 ▸); program(s) used to refine structure: *SHELXL97* (Sheldrick, 2008 ▸); molecular graphics: *Mercury* (Macrae *et al.*, 2008 ▸); software used to prepare material for publication: *SHELXL97* and *PLATON* (Spek, 2009 ▸).

## Supplementary Material

Crystal structure: contains datablock(s) global, I. DOI: 10.1107/S2056989015010087/su5142sup1.cif

Structure factors: contains datablock(s) I. DOI: 10.1107/S2056989015010087/su5142Isup2.hkl

Supporting information file. DOI: 10.1107/S2056989015010087/su5142Isup3.cml

**Fig. 1.**
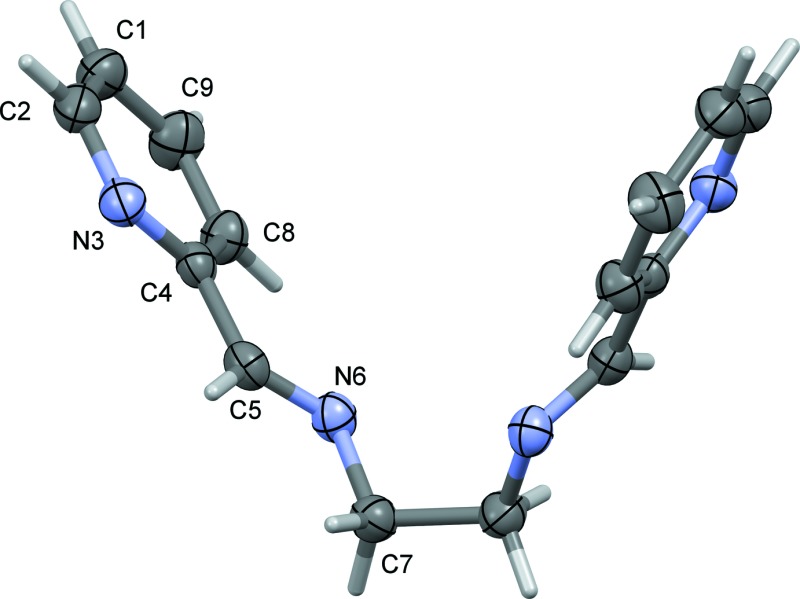
View of the molecular structure of the title compound, with atom labelling. Displacement ellipsoids are drawn at the 50% probability level. Unlabelled atoms are related to the labelled atoms by twofold rotation symmetry (symmetry code: -x + 1, y, -z - 1/2).

Click here for additional data file.x y z . DOI: 10.1107/S2056989015010087/su5142fig1.tif
View of the mol­ecular structure of the title compound, with atom labelling. Displacement ellipsoids are drawn at the 50% probability level. Unlabelled atoms are related to the labelled atoms by twofold rotation symmetry (symmetry code: −*x* + 1, *y*, −*z* − 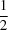).

CCDC reference: 1402701

Additional supporting information:  crystallographic information; 3D view; checkCIF report

## supplementary crystallographic information

### S1. Structural commentary

Schiff bases derived from pyridine­carbaldehydes have received considerable inter­est in synthetic chemistry (Marjani *et al.*, 2009). 1,2-di­amine-pyridine derived Schiff base bidentate or polydentate chelating ligand towards metal centers draw major attraction towards synthesis and medical application (Warad *et al.*, 2014). It is still challenging to design and rationally synthesize ligands with unique structures and functions.

### S2. Synthesis and crystallization

To a solution of pyridine-2-carbaldehyde (1 mmol) dissolved in 10 ml of absolute ethanol was added drop wise ethane-1,2-di­amine (1 mmol) in 5 ml of absolute ethanol under constant stirring for 10 min. The mixture was refluxed for 4 h and then concentrated under reduced pressure. The title compound was precipitated by the addition of 50 ml of n-hexane. It was filtered off, washed three times with 80 ml of distilled water then with di­ethyl ether to give the title compound (yield: 86%). Single crystals suitable for X-ray analysis were obtained within two days by slow evaporation of a solution in di­chloro­methane.

### S3. Refinement

Crystal data, data collection and structure refinement details are summarized in Table 1. The H atoms were fixed geometrically (C—H = 0.93 – 0.97 Å) and allowed to ride on their parent atoms with *U*_iso_(H) = 1.2*U*_eq_(C).

### Crystal data

**Table:**

C_14_H_14_N_4_	*F*(000) = 504
*M**_r_* = 238.29	*D*_x_ = 1.238 Mg m^−^^3^
Monoclinic, *C*2/*c*	Cu *K*α radiation, λ = 1.54178 Å
Hall symbol: -C 2yc	Cell parameters from 881 reflections
*a* = 19.347 (5) Å	θ = 5.4–63.8°
*b* = 5.9339 (12) Å	µ = 0.61 mm^−^^1^
*c* = 13.165 (2) Å	*T* = 296 K
β = 122.266 (8)°	Block, colourless
*V* = 1278.0 (5) Å^3^	0.30 × 0.27 × 0.25 mm
*Z* = 4	

### Data collection

**Table:**

Bruker X8 Proteum diffractometer	933 independent reflections
Radiation source: Rotating Anode	881 reflections with *I* > 2σ(*I*)
Graphite monochromator	*R*_int_ = 0.015
Detector resolution: 18.4 pixels mm^-1^	θ_max_ = 63.8°, θ_min_ = 5.4°
φ and ω scans	*h* = −8→21
Absorption correction: multi-scan (*SADABS*; Bruker, 2013)	*k* = −6→5
*T*_min_ = 0.837, *T*_max_ = 0.862	*l* = −15→12
1539 measured reflections	

### Refinement

**Table:**

Refinement on *F*^2^	Primary atom site location: structure-invariant direct methods
Least-squares matrix: full	Secondary atom site location: difference Fourier map
*R*[*F*^2^ > 2σ(*F*^2^)] = 0.043	Hydrogen site location: inferred from neighbouring sites
*wR*(*F*^2^) = 0.120	H-atom parameters constrained
*S* = 1.05	*w* = 1/[σ^2^(*F*_o_^2^) + (0.0763*P*)^2^ + 0.2763*P*] where *P* = (*F*_o_^2^ + 2*F*_c_^2^)/3
933 reflections	(Δ/σ)_max_ < 0.001
82 parameters	Δρ_max_ = 0.10 e Å^−^^3^
0 restraints	Δρ_min_ = −0.10 e Å^−^^3^

### Special details

**Table:**

Geometry. Bond distances, angles *etc*. have been calculated using the rounded fractional coordinates. All su's are estimated from the variances of the (full) variance-covariance matrix. The cell e.s.d.'s are taken into account in the estimation of distances, angles and torsion angles
Refinement. Refinement on *F*^2^ for ALL reflections except those flagged by the user for potential systematic errors. Weighted *R*-factors *wR* and all goodnesses of fit *S* are based on *F*^2^, conventional *R*-factors *R* are based on *F*, with *F* set to zero for negative *F*^2^. The observed criterion of *F*^2^ > σ(*F*^2^) is used only for calculating -*R*-factor-obs *etc*. and is not relevant to the choice of reflections for refinement. *R*-factors based on *F*^2^ are statistically about twice as large as those based on *F*, and *R*-factors based on ALL data will be even larger.

### Fractional atomic coordinates and isotropic or equivalent isotropic displacement parameters (Å^2^)

**Table:**

	*x*	*y*	*z*	*U*_iso_*/*U*_eq_	
N3	0.69126 (7)	0.50665 (18)	0.02615 (10)	0.0576 (4)	
N6	0.55349 (7)	0.05345 (19)	−0.11311 (9)	0.0542 (4)	
C1	0.67191 (10)	0.7085 (3)	0.16610 (14)	0.0703 (6)	
C2	0.70819 (9)	0.6792 (2)	0.10143 (14)	0.0634 (5)	
C4	0.63482 (8)	0.3583 (2)	0.01373 (11)	0.0478 (4)	
C5	0.61592 (8)	0.1747 (2)	−0.07264 (11)	0.0495 (4)	
C7	0.54261 (9)	−0.1287 (2)	−0.19397 (13)	0.0585 (5)	
C8	0.59615 (9)	0.3750 (2)	0.07686 (12)	0.0591 (5)	
C9	0.61501 (11)	0.5541 (3)	0.15343 (14)	0.0728 (6)	
H1	0.68570	0.83080	0.21760	0.0840*	
H2	0.55830	0.26720	0.06750	0.0710*	
H4	0.74660	0.78490	0.11040	0.0760*	
H5	0.65170	0.14760	−0.09810	0.0590*	
H7	0.58250	−0.11360	−0.21680	0.0700*	
H8	0.55230	−0.27160	−0.15260	0.0700*	
H9	0.58950	0.57050	0.19620	0.0870*	

### Atomic displacement parameters (Å^2^)

**Table:**

	*U*^11^	*U*^22^	*U*^33^	*U*^12^	*U*^13^	*U*^23^
N3	0.0509 (8)	0.0571 (7)	0.0602 (7)	0.0007 (5)	0.0265 (6)	0.0064 (5)
N6	0.0501 (8)	0.0609 (7)	0.0466 (6)	0.0031 (5)	0.0225 (5)	0.0005 (5)
C1	0.0714 (11)	0.0636 (9)	0.0601 (9)	−0.0011 (7)	0.0246 (8)	−0.0102 (7)
C2	0.0554 (10)	0.0564 (9)	0.0628 (9)	−0.0038 (6)	0.0212 (7)	0.0035 (6)
C4	0.0410 (8)	0.0514 (7)	0.0430 (7)	0.0068 (5)	0.0171 (6)	0.0090 (5)
C5	0.0463 (8)	0.0551 (8)	0.0473 (7)	0.0085 (6)	0.0251 (6)	0.0079 (5)
C7	0.0600 (9)	0.0532 (8)	0.0540 (8)	0.0048 (6)	0.0249 (7)	−0.0001 (6)
C8	0.0561 (9)	0.0674 (9)	0.0542 (8)	−0.0052 (7)	0.0298 (7)	−0.0034 (6)
C9	0.0743 (12)	0.0866 (11)	0.0628 (9)	−0.0047 (8)	0.0401 (9)	−0.0136 (8)

### Geometric parameters (Å, º)

**Table:**

N3—C2	1.3384 (18)	C8—C9	1.373 (2)
N3—C4	1.343 (2)	C1—H1	0.9300
N6—C5	1.254 (2)	C2—H4	0.9300
N6—C7	1.4514 (18)	C5—H5	0.9300
C1—C2	1.373 (3)	C7—H7	0.9700
C1—C9	1.372 (3)	C7—H8	0.9700
C4—C5	1.4730 (18)	C8—H2	0.9300
C4—C8	1.387 (2)	C9—H9	0.9300
C7—C7^i^	1.516 (2)		
			
C2—N3—C4	116.96 (15)	N3—C2—H4	118.00
C5—N6—C7	117.93 (15)	C1—C2—H4	118.00
C2—C1—C9	118.85 (16)	N6—C5—H5	119.00
N3—C2—C1	123.50 (16)	C4—C5—H5	119.00
N3—C4—C5	115.43 (14)	N6—C7—H7	109.00
N3—C4—C8	122.94 (12)	N6—C7—H8	109.00
C5—C4—C8	121.62 (13)	H7—C7—H8	108.00
N6—C5—C4	122.55 (15)	C7^i^—C7—H7	109.00
N6—C7—C7^i^	111.74 (13)	C7^i^—C7—H8	109.00
C4—C8—C9	118.56 (16)	C4—C8—H2	121.00
C1—C9—C8	119.17 (19)	C9—C8—H2	121.00
C2—C1—H1	121.00	C1—C9—H9	120.00
C9—C1—H1	121.00	C8—C9—H9	120.00
			
C2—N3—C4—C5	178.17 (12)	N3—C4—C5—N6	−164.26 (12)
C2—N3—C4—C8	−1.6 (2)	C5—C4—C8—C9	−178.16 (14)
C4—N3—C2—C1	0.9 (2)	C8—C4—C5—N6	15.5 (2)
C7—N6—C5—C4	−177.50 (11)	N3—C4—C8—C9	1.6 (2)
C5—N6—C7—C7^i^	−131.18 (14)	N6—C7—C7^i^—N6^i^	73.41 (17)
C9—C1—C2—N3	−0.2 (3)	C4—C8—C9—C1	−0.8 (2)
C2—C1—C9—C8	0.1 (3)		

Symmetry code: (i) −*x*+1, *y*, −*z*−1/2.
