# Supplementary figures and images for: Crystal structure of (1E,1′E)-N,N′-(ethane-1,2-di­yl)bis­[(pyridin-2-yl)methanimine]
Source: Acta Crystallogr E Crystallogr Commun. 2015 May 30;71(Pt 6):o431. doi: 10.1107/S2056989015010087 (PMC4459302; doi:10.1107/S2056989015010087)

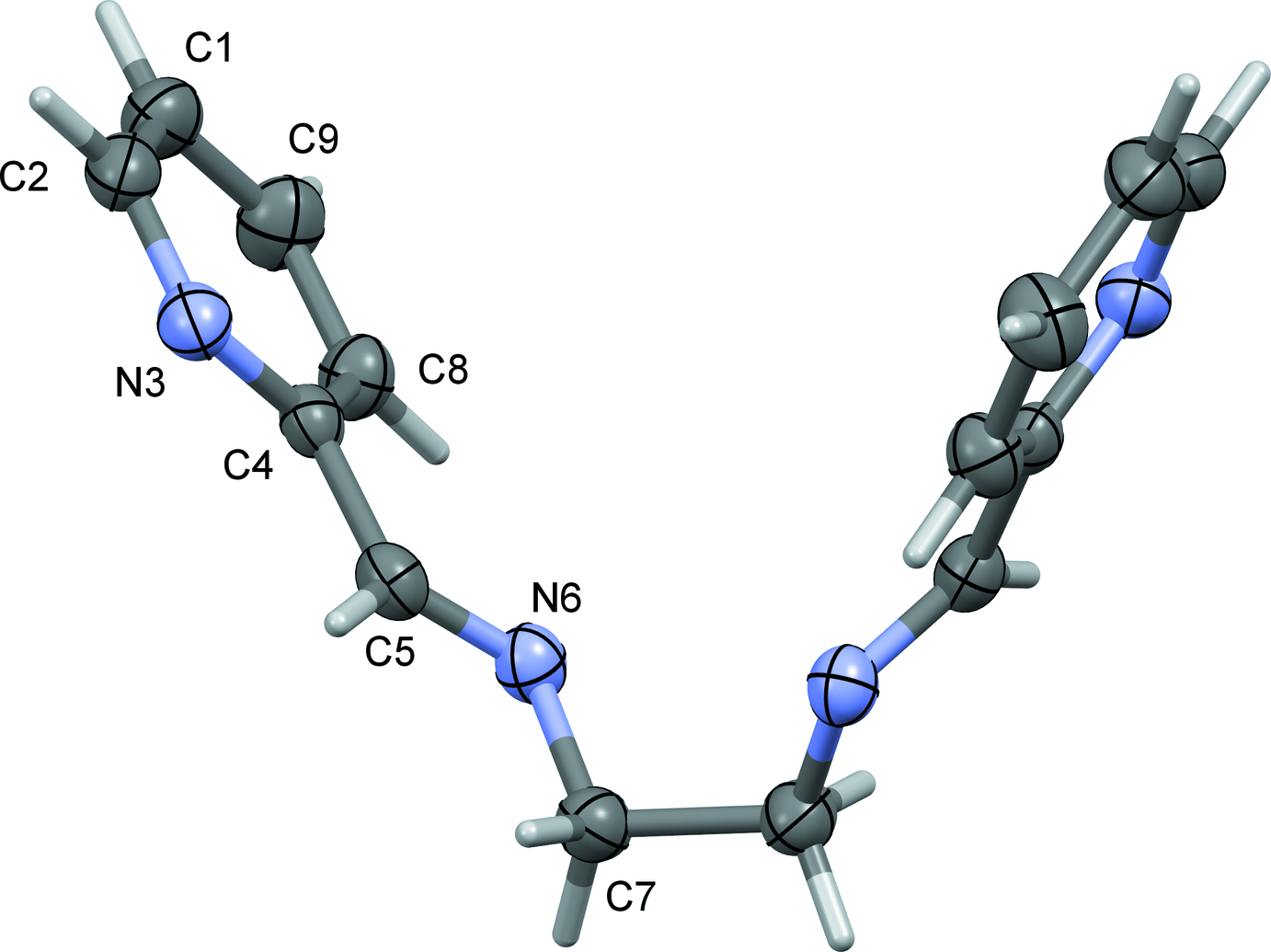

Supplement: Supplementary file 4 [file e-71-0o431-fig1.tif]
